# Bioactive Polyphenols from the Methanol Extract of *Cnicus arvensis* (L.) Roth Demonstrated Antinociceptive and Central Nervous System Depressant Activities in Mice

**DOI:** 10.1155/2015/794729

**Published:** 2015-01-14

**Authors:** Mahmudur Rahman, Amina Khatun, Mst. Luthfun Nesa, Hemayet Hossain, Ismet Ara Jahan

**Affiliations:** ^1^Natural Product Chemistry and Pharmacology Research Laboratory, Faculty of Health Sciences, Department of Pharmacy, Northern University Bangladesh, Dhaka 1205, Bangladesh; ^2^Phytochemistry and Pharmacology Research Laboratory, Department of Pharmacy, Manarat International University, Dhaka 1216, Bangladesh; ^3^Department of Pharmacy, Atish Dipankar University of Science and Technology, Dhaka 1213, Bangladesh; ^4^Chemical Research Division, BCSIR Laboratories, Bangladesh Council of Scientific & Industrial Research, Dhaka 1205, Bangladesh

## Abstract

*Cnicus arvensis* is used by many ethnic groups for inflammation, pain, and other ailments. In this study, reducing sugar, carbohydrate, alkaloid, steroid, tannin, flavonoid, and saponin groups were identified using standard chromogenic method. In high-performance liquid chromatography, vanillic acid and epicatechin were identified in the extract. Antinociceptive test by acetic acid induced writhing inhibition resulted 43.17 and 95.08% inhibition for 100 and 200 mg/kg body weight, comparing with standard diclofenac Na with 74.86% inhibition for 25 mg/kg body weight. In formalin induced paw licking test for antinociceptive activity, the extract inhibited 69.87 and 75.55% licking for 150 and 300 mg/kg body weight comparing with the inhibition (68.56%) of diclofenac Na for 10 mg/kg body weight at first phase. At late phase, the extract showed 73.12 and 87.46% licking comparing with licking inhibition (71.69%) by diclofenac Na at the same dose. In open field test for CNS depressant activity, the extract showed depression of locomotor activity for 150 and 300 mg/kg body weight comparing with diazepam for 10 mg/kg body weight. All results were statistically significant (*P* < 0.01). The identified polyphenols are reputed for antinociceptive and CNS depressant activity. The present findings support the use of this plant in pain.

## 1. Introduction

*Cnicus arvensis* (L.) Scop. Roth. (Hoffm.), synonym:* Cirsium arvense *(L.) Scop. (Bengali name: Biralkanta; milk thistle, common cephalonoplos, and creeping thistle in English; family: Asteraceae), is an erect herb which is found in Bangladesh [1]. The flower heads are solitary, purple, and petals connate in slender tabular corollas. The plant is used in the treatment of cirrhosis, diabetes, excessive menstruation, gout, gastritis, liver cancer, jaundice, lipoma (tumor), and scabies [1]. The rhizome is popularly used for its astringent properties [2]. It is used as cholagogic and diuretic [3]. Medicinal and toxic properties of* C. arvensis *are reported in the books of ethnopharmacological relevance [1, 4, 5]. Native Americans (Meskwaki) use the root to disguise taste of medicine. Thompson nation dried roots, later scraped away dirt and skin and cooked in stews and soups. Cherokee used infusion of leaves for neuritis and neuralgia. Poultice of the plant is used as analgesic. Abenaki nation used a decoction of roots as anthelminthic for deworming children. Ojibwa used whole plant decoction to cleanse bowels. Mohegan and Montagnals used decoction to treat tuberculosis. Iroquois used infusion of root as mouth wash. The Chinese use thistle teas to treat appendicitis, internal bleeding, and inflammations [6]. The whole plant is tonic, emetic, anthelmintic, diaphoretic, vermifuge, and emmenagogue and is used for epistaxis, pulmonary troubles, tuberculosis, and pyogenic infections. Leaf juice is used on wounds. Roots are diuretic and are used in urinary complaints; aqueous extract of roots is given for the treatment of liver disorders [7]. The plant contains bitter ethereal compounds, volatile oils, and tannin [3]. Cytotoxic property of this plant has been reported [6].

A food supplement (milk thistle 3000 mg) in the tablet form is marketed by Simply Supplements, Le Bourg, Forest, Guernsey, and claimed to contain the key ingredient of milk thistle, silymarin, a flavonoid compound extracted from the seeds. Though this plant is a common species and is well documented for folk uses in the reference ethnopharmacological compendia, it is yet not extensively studied. To the best of our search, no previous phytochemical and pharmacological investigation was conducted on this plant till now.

The current study was designed to provide scientific evidence for its use as a conventional folk remedy by examining the antinociceptive and CNS depressant properties.

## 2. Materials and Methods

### 2.1. Collection and Identification of Plant Material

The aerial parts of* Cnicus arvensis *were collected from Khulna University Campus, Khulna, Bangladesh, in March 2013 (coordinate: 22.35°N 89.08°E). The samples of the plant were mounted on paper and the species was taxonomically confirmed by Sarder Nasir Uddin, Principle Scientific Officer, Bangladesh National Herbarium, Mirpur, Dhaka. A voucher specimen of the plant has been deposited and preserved in the library of the same institution for further collection and reference (accession number: DACB-31,544).

### 2.2. Preparation of Methanol Extract

The collected plants were separated from undesirable materials and shade-dried for two weeks. The shade-dried plant materials were ground into a coarse powder with the help of a suitable grinder (capacitor start motor, Wuhu Motor Factory, China). The powdered sample was stored in an airtight container and kept in a cool, dark, and dry place until analysis commenced. About 500 g of powered material was taken in a clean, flat-bottomed glass container and soaked in 1900 mL of methanol. The container along with its contents was sealed and kept for a period of 15 days with occasional shaking or stirring [8]. The whole mixture then underwent a coarse filtration by cotton. It was then filtered through Whatman filter paper (Bibby RE200, Sterilin Ltd., UK). The filtrate (ethanol extract) thus obtained was evaporated at low temperature (40°C) and reduced pressure using a rotary vacuum evaporator (R-210, Buchi, Switzerland). It yielded a gummy concentrate of brownish black color. The gummy concentrate was then freeze dried (D-37520, Martin Christ, Germany) and was obtained as crude extract of methanol. It yielded a 5 g concentrate (10% yields) of plant powder.

### 2.3. Test Animals, Chemicals, and Drugs

Swiss-albino mice,* Mus musculus*, of either sex of 3-4 weeks of age, weighing 20–25 g of either sex were purchased from the Animal Research Branch of the International Centre for Diarrhoeal Disease and Research, Bangladesh (ICDDR, B), and were used for* in vivo *antinociceptive and CNS depressant screening. The animals were housed in plastic cages in standard environmental conditions at the animal house in Department of Pharmacy, Northern University Bangladesh for adaptation after their purchase under standard laboratory conditions (relative humidity 55–65%, room temperature 25.0 ± 2.0°C, and 12 h light-dark cycle). They were fed with standard vital feed (ICDDR, B formulated grower pelletized) and water was available* ad libitum* throughout the period of acclimatization. The research was carried out according to the National Institutes of Health (NIH) Guide for the Care and Use of Laboratory Animals and Organization for Economic Cooperation and Development (OECD) guidelines. The experimental protocol was approved by the Animal Ethics Committee, Northern University Bangladesh (Ref.: NUB/Pharm/AEC-2013).

Methanol (≥99.5%, Merck KGaA, Darmstadt, Germany) was used as solvent for maceration of the plant material. Lead acetate, potassium dichromate, ferric chloride, hydrochloric acid, sulphuric acid, Mayer's reagent, Dragendorff's reagent, Wagner's reagent, Hager's reagent, Molisch's reagent, Benedict's reagent, and Fehling's solutions were purchased from Himedia (Mumbai, India) for preliminary phytochemical screening. The standard drugs diclofenac Na used for antinociceptive screening and diazepam for CNS depressant activity test were purchased from Square Pharmaceuticals Ltd., Bangladesh. Tween 80 used as suspending agent for the extracts was purchased from Merck (Darmstadt, Germany). Acetic acid, the pain inducer, was also purchased from Himedia (Mumbai, India). Formalin (Ranbaxy (Rankem)) and carboxymethyl cellulose for antinociceptive test were purchased from the local drug market. Gallic acid (GA), (+)-catechin hydrate (CH), vanillic acid (VA), caffeic acid (CA), (−)-epicatechin (EC),* p*-coumaric acid (PCA), rutin hydrate (RH), ellagic acid (EA), and quercetin (QU) were purchased from Sigma–Aldrich (St. Louis, MO, USA). HPLC grade acetonitrile, methanol, acetic acid, and ethanol were obtained from Merck (Darmstadt, Germany).

### 2.4. Instruments, Equipment, and Software

Electronic balance (serial number: 1508, OHAUS, Germany) and vortex mixer (VM-2000, 220 V, Digisystem Laboratory Instruments Inc., Taiwan) were used for this study. Chromatographic analyses using high performance liquid chromatography (HPLC) of the extract were carried out on Thermo Scientific Dionex Ultimate 3000 Rapid Separation LC (RSLC) systems (Thermo Fisher Scientific Inc., MA, USA), coupled to a quaternary rapid separation pump (LPG-3400RS), Ultimate 3000RS autosampler (WPS-3000), and rapid separation diode array detector (DAD-3000RS). Phenolic compounds were separated on an Acclaim C18 (4.6 × 250 mm; 5 *μ*m) column (Dionex, USA) which was controlled at 30°C using a temperature controlled column compartment (TCC-3000). Data acquisition, peak integration, and calibrations were performed with Dionex Chromeleon software (Version 6.80 RS 10).

### 2.5. Test for Different Chemical Groups

The phytochemical screening of the crude methanol extract was carried out by standard chromogenic reagents: lead acetate, potassium dichromate, ferric chloride, hydrochloric acid, sulphuric acid, Mayer's reagent, Dragendorff's reagent, Wagner's reagent, Hager's reagent, Molisch reagent, Benedict's reagent, and Fehling's solutions to detect steroids, alkaloids, carbohydrates, gums, flavonoids, saponins, tannins, and reducing sugars using slight modification of standard protocol [9]. The color intensity or the precipitate formation was used as analytical responses to these tests. 10% (w/v) solution of the extract in methanol was used for each of the above tests.

### 2.6. High Performance Liquid Chromatography (HPLC) Analysis of the Extract

#### 2.6.1. High Performance Liquid Chromatography (HPLC) System

Chromatographic analyses were carried out on a Thermo Scientific Dionex Ultimate 3000 Rapid Separation LC (RSLC) systems (Thermo Fisher Scientific Inc., MA, USA), coupled to a quaternary rapid separation pump (LPG-3400RS), Ultimate 3000RS autosampler (WPS-3000), and rapid separation diode array detector (DAD-3000RS). Phenolic compounds were separated on an Acclaim C18 (4.6 × 250 mm; 5 *μ*m) column (Dionex, USA). It was controlled at 30°C using a temperature controlled column compartment (TCC-3000). Data acquisition, peak integration, and calibrations were performed with Dionex Chromeleon software (Version 6.80 RS 10).

#### 2.6.2. Chromatographic Conditions

The phenolic compositions of the methanol extract of* Cnicus arvensis *were determined by HPLC, as described by Sarunya and Sukon [10] with some modifications. The mobile phase consisted of acetonitrile (solvent A), acetic acid solution of pH 3.0 (solvent B), and methanol (solvent C). The system was run with the following gradient elution program: 0 min, 5%A/95%B; 10 min, 10%A/80%B/10%C; 20 min, 20%A/60%B/20%C; and 30 min, 100%A. There was a 5 min postrun at initial conditions for equilibration of the column. The flow rate was kept constant throughout the analysis at 1 mL/min and the injection volume was 20 *μ*L. For UV detection, the wavelength program was optimized to monitor phenolic compounds at their respective maximum absorbance wavelengths as follows: *λ* 280 nm held for 18.0 min, changed to *λ* 320 nm and held for 6 min, and finally changed to *λ* 380 nm and held for the rest of the analysis, and the diode array detector was set at an acquisition range from 200 nm to 700 nm. The detection and quantification of GA, CH, VA, CA, and EC were done at 280 nm, of PCA, RH, and EA at 320 nm, and of MC, QU, and KF at 380 nm, respectively.

#### 2.6.3. Standard and Sample Preparation

A stock standard solution (100 *μ*g/mL) of each phenolic compound was prepared in methanol by weighing out approximately 0.0050 g of the analyte into 50 mL volumetric flask. The mixed standard solution was prepared by dilution of the mixed stock standard solutions in methanol to give a concentration of 5 *μ*g/mL for each polyphenol except (+)-catechin hydrate, caffeic acid, rutin hydrate (4 *μ*g/mL), and quercetin (3 *μ*g/mL). All standard solutions were stored in the dark at 5°C and were stable for at least three months.

The calibration curves of the standards were made by a dilution of the stock standards (five sets of standard dilutions) with methanol to yield 1.0–5.0 *μ*g/mL for GA, CH, VA, EC, PCA, EA, MC, and KF, 0.5–4.0 *μ*g/mL for CH, CA, and RH, and 0.25–3.0 *μ*g/mL for QU. The calibration curves were constructed from chromatograms as peak area versus concentration of standard.

A solution of methanol extract of* C. arvensis *at a concentration of 5 mg/mL was prepared in methanol by vortex mixing (Branson, USA) for 30 min. The samples were stored in the dark at low temperature (5°C). Spiking the sample solution with phenolic standards was done for additional identification of individual polyphenols.

Prior to HPLC analysis, all solutions (mixed standards, sample, and spiked solutions) were filtered through 0.20 *μ*m nylon syringe filter (Sartorius, Germany) and then degassed in an ultrasonic bath (Hwashin, Korea) for 15 min.

#### 2.6.4. Peak Characterization and Quantification

The compounds were identified by comparing with standards of each identified compound using the retention time and the absorbance spectrum profile and also by running the samples after the addition of pure standards. Quantification was performed by establishing calibration curves for each compound determined, using the standards. Linear calibration curves for standards (peak area versus concentration) were constructed with *R* ^2^ exceeding 0.995. Data are reported as means ± standard deviations of triplicate independent analyses.

### 2.7. Evaluation of Antinociceptive Activity

#### 2.7.1. Acetic Acid Induced Writhing Method

The method of Hossain et al. [11] was adopted with minor modification for the antinociceptive activity study for the crude methanol extract of* Cnicus arvensis *using the acetic acid induced writhing model in mice. The animals were divided into control, positive control, and test groups with six mice in each group. The animals of test groups received the plant extract at the dose of 100 and 200 mg/kg body weight each. The positive control group was treated with diclofenac Na (standard drug) at a dose of 25 mg/kg body weight and the vehicle control group was treated with 1% tween 80 in distilled water at a dose of 10 mL/kg body weight. Test samples, standard drug, and control vehicle were administered orally 30 minutes before intraperitoneal administration of 0.7% (v/v) acetic acid solution (0.1 mL/10 g body weight) to induce abdominal contractions or writhing. Five minutes after the administration of acetic acid, the number of writhing (constriction of abdomen, turning of trunk, and extension of hind legs) for each animal was counted for 15 minutes. The number of writhing in the control was taken as 100% and percent inhibition was calculated as follows:

(1) %  Inhibition  of  writhing=100−treated  meancontrol  mean×100.

#### 2.7.2. Formalin Induced Paw Licking in Mice

The antinociceptive activity of the test samples was also determined using the formalin induced paw licking test in mice [12]. Formalin (0.2 mL of 5% v/v freshly prepared formalin solution prepared in distilled water) was used as oedematogenic agent [13]. 20 *μ*L of 5% formalin was injected into the dorsal surface of the right hind paw of all the group of mice 60 minutes after administration of test samples and 30 minutes after administration of diclofenac Na (10 mg/kg body weight, i.p.). Control group received only 5% formalin. The mice were observed for 30 minutes after the injection of formalin, and the amount of time spent licking the injected hind paw was recorded. The first 5 minutes of postformalin injection is referred to as the early phase and the period between 15 and 30 minutes as the late phase. The total time spent licking or biting the injured paw (pain behavior) was measured with a stopwatch.

### 2.8. Open Field Test

The open field test was carried out as described by Gupta et al. [14]. The animals were divided into control, standard, and test groups (*n* = 6 per group). The control group received vehicle (1% tween 80 in water at the dose of 10 mL/kg p.o.). The test group received the crude extract (at the doses of 150 and 300 mg/kg body weight p.o.) and standard group received diazepam at the dose of 1 mg/kg body weight orally. The animals were placed on the floor of an open field (100 cm × 100 cm × 40 cm h) divided into a series of squares. The number of squares visited by each animal was counted for 3 minutes on 0, 30, 60, 90, and 120 minutes during the study period.

### 2.9. Statistical Analysis

Data were presented as mean ± SEM, and comparisons were made using one-way analysis of variance (ANOVA). Statistical differences between control and treated groups were compared by Student's *t*-test. The differences were considered significant at *P* < 0.01.

## 3. Results

### 3.1. Phytochemical Analysis

Results of different chemical tests on the methanol extract of* Cnicus arvensis* aerial parts showed the presence of reducing sugar, carbohydrate, alkaloids, steroids, flavonoids, tannin, and saponins (Table 1).

### 3.2. Phenolic Contents by HPLC Analysis

**Figure 1:**
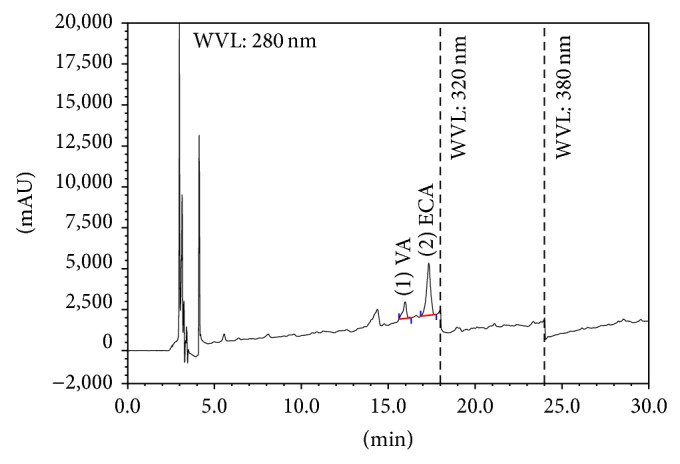
HPLC chromatogram of* Cnicus arvensis* extract. Peaks: (1) vanillic acid; (2) (−)-epicatechin.

Identification and quantification of individual phenolic compounds in the methanol extract of* Cnicus arvensis *were analysed by HPLC. The chromatographic separations of polyphenols in methanol extract are shown in Figure 1. The content of each phenolic compound was calculated from the corresponding calibration curve and presented as the mean of five determinations as shown in Table 2.

The experimental results indicated that methanol extract of* C. arvensis *contained moderately high concentration of vanillic acid and (−)-epiquercetin (2.06 mg and 39.14 mg per 100 g of dry extract weight, resp.).

### 3.3. Evaluation of Antinociceptive Activity

Antinociceptive activity of the methanol extract of* Cnicus arvensis *was tested on acetic acid induced writhing and formalin induced paw licking in mice.

#### 3.3.1. Acetic Acid Induced Writhing Method

At the dose of 100 and 200 mg/kg of body weight, the plant extract produced 43.17 and 95.08% inhibition of writhing, respectively, in test animals in both dose-dependent manners in the acetic acid induced writhing test (Table 3). The results were statistically significant (*P* < 0.01) and were comparable to the standard drug (diclofenac Na) which showed 74.86% inhibition of writhing at a dose of 25 mg/kg body weight.

#### 3.3.2. Formalin Induced Paw Licking Activity

*C. arvensis *extract significantly (*P* < 0.01) suppressed the licking activity in either phase of the formalin induced pain in mice inhibiting 69.87 and 75.55% of paw licking, respectively, at the dose of 150 and 300 mg/kg of body weight in comparison with standard diclofenac Na (68.56% licking inhibition) at the dose of 10 mg/kg body weight in the first phase. The extract inhibited 73.12 and 87.46% of licking and standard diclofenac Na inhibited 71.69% licking at the previously stated dose (Table 4).

### 3.4. Evaluation of CNS Depressant Activity by Open Field Test

In the open field test,* Cnicus arvensis *extract exhibited a decrease in the movements of the test animals at all tested dose levels. The results were statistically significant (*P* < 0.01) for all doses with a dose-dependent response (Table 5).

## 4. Discussion

This study revealed the presence of reducing sugars, carbohydrates, alkaloids, steroids, tannins, flavonoids, and saponins as major secondary metabolites. Previous studies suggested that secondary metabolites of plants, namely, alkaloids, tannins, many terpenoids, and flavonoids, have been contributing to reducing both neurogenic and nonneurogenic pains as well as exerting tranquilizing, CNS depressing, and other neuropharmacological effects [15–19]. The acetic acid induced writhing test is a chemical method used to induce profound pain of peripheral origin for the evaluation of analgesic drugs. Analgesic activity of the test compound is inferred from decrease in the frequency of writhing. The manifestations of abdominal writhing in mice are described as an arching of back, extension of hind limbs, and contraction of abdominal musculature [20]. The extract inhibited the writhing significantly at dose-dependent manner. The biphasic model of formalin induced paw licking is represented by neurogenic (0–5 minutes) and inflammatory pain (15–30 minutes) [21]. The formalin test does not require sophisticated equipment and is very easy to carry out. Since the test assesses the response of the animal to moderate, continuous pain, it has been proposed as a more valid model for clinical pain compared to tests using phasic thermal or mechanical stimuli. As the test is based on the observation of spontaneous nociceptive behaviors following injection of formalin in the experimental animal hind paw, intersubject variability is quite high especially for the licking response [22]. The inhibition of paw licking in both phases by the plant extract indicates both antinociceptive and anti-inflammatory effects.

Plants containing vanillic acid and epicatechin have been reported previously for antinociceptive activity [23, 24]. In our study, the plant exhibited significant antinociceptive activities in both tests. As the plant contains both vanillic acid and epicatechin, the plant may be employed in a wide range of pain.

Extract of* C. arvensis *decreased locomotor activity which indicates its CNS depressant activity. Gamma-aminobutyric acid (GABA) is the major inhibitory neurotransmitter in the central nervous system [25]. Earlier investigation has also demonstrated that phenolic acids such as epicatechin were found to be ligands for the GABA_A_ receptors in the central nervous system [26], which led to assumption that they can act as benzodiazepine-like molecules.

## 5. Conclusion

HPLC analysis showed the presence of vanillic acid and epicatechin in the plant extract, so it might be possible that these phytoconstituents are responsible for its antinociceptive and CNS depressant activities. On the other hand, this study supports the ethnomedicinal use of this plant in pain and inflammation. Mechanism of action of the compounds responsible for antinociceptive and CNS depressant activities may be identified on the basis of this study.

## Figures and Tables

**Table 1:** Results of phytochemical screening of *Cnicus arvensis* extract.

Test for phytochemical group	Reagent	Extract of *Cnicus arvensis *
Reducing sugar	Fehling's test	+
	Benedict's test	+

Carbohydrate	Barfoed's test	+

Alkaloid	Mayer's test	+
	Dragendorff's test	+

Steroid	Salkowski's test	+
	Liebermann Burchard test	+

Tannin	Ferric chloride test	+

Gum	Molisch's test	−

Flavonoid	Shinoda test	+
	Alkaline reagent test	+

Saponin	Frothing test	+

+ indicates presence and − indicates absence.

**Table 2:** Contents of polyphenolic compounds in *Cnicus arvensis *extract.

Polyphenolic compound	Methanol extract of *Cnicus arvensis *	
	Content (mg/100 g of dry extract)	% RSD
VA	2.06	0.15
EC	39.14	1.79

*n* = 3.

**Table 3:** Effects of *Cnicus arvensis *extract on acetic acid induced writhing in mice.

Animal group	Treatment	Writhing (mean ± SEM)	% inhibition
Group I	1% tween solution in water	30.5 ± 0.43	0
Group II	Diclofenac Na (25 mg/kg bd. wt.)	7.67 ± 1.13^*^	74.86
Group III	Plant extract (100 mg/kg bd. wt.)	17.33 ± 3.64^*^	43.17
Group IV	Plant extract (200 mg/kg bd. wt.)	1.50 ± 0.76^*^	95.08

bd. wt. = body weight; SEM = standard error of mean; *n* = 6; ^*^ *P* < 0.01.

**Table 4:** Effects of *Cnicus arvensis *extract on formalin induced paw licking test in mice.

Groups	First phase		Late phase	
	Duration of licking	% inhibition	Duration of licking	% inhibition
Control 1% tween 80 in water	38.17 ± 1.27	—	46.5 ± 0.99	—
Positive control 10 mg/kg bd. wt.	12.00 ± 0.70^*^	68.56	13.17 ± 0.48^*^	71.69
Plant extract 150 mg/kg bd. wt.	11.50 ± 2.42^*^	69.87	12.50 ± 2.72^*^	73.12
Plant extract 300 mg/kg bd. wt.	9.33 ± 1.35^*^	75.55	5.83 ± 1.35^*^	87.46

bd. wt. = body weight; values are mean ± SEM; *n* = 6; ^*^ *P* < 0.01.

**Table 5:** Effects of *Cnicus arvensis *extract on open field test in mice.

Groups	Responses at				
	min 0	min 30	min 60	min 90	min 120
Control	115.67 ± 2.81	115.17 ± 1.20	110.50 ± 0.99	98.67 ± 1.50	105.00 ± 1.57
Positive control	105.67 ± 2.01^*^	73.67 ± 3.64^*^	51.17 ± 1.40^*^	24.50 ± 2.80^*^	13.17 ± 1.25^*^
Plant extract of 150 mg/kg bd. wt.	101.5 ± 4.93	128.67 ± 8.62	75.00 ± 8.73^*^	30.00 ± 5.59^*^	33.00 ± 6.76^*^
Plant extract of 300 mg/kg bd. wt.	206.5 ± 9.79^*^	159.17 ± 2.80^*^	105.50 ± 3.96^*^	101.50 ± 3.27^*^	74.17 ± 6.76^*^

bd. wt. = body weight; values are mean ± SEM; *n* = 6; ^*^ *P* < 0.01.
